# RSPO3-LGR4 Regulates Osteogenic Differentiation Of Human Adipose-Derived Stem Cells Via ERK/FGF Signalling

**DOI:** 10.1038/srep42841

**Published:** 2017-02-21

**Authors:** Min Zhang, Ping Zhang, Yunsong Liu, Longwei Lv, Xiao Zhang, Hao Liu, Yongsheng Zhou

**Affiliations:** 1 Department of Prosthodontics, Peking University School and Hospital of Stomatology, Beijing, 100081, China; 2 National Engineering Lab for Digital and Material Technology of Stomatology, Beijing Key Laboratory of Digital Stomatology, Peking University School and Hospital of Stomatology, Beijing, 100081, China; 3 Central Laboratory, Peking University School and Hospital of Stomatology, Beijing, 100081, China

## Abstract

The four R-spondins (RSPOs) and their three related receptors, LGR4, 5 and 6, have emerged as a major ligand-receptor system with critical roles in development and stem cell survival. However, the exact roles of the RSPO-LGR system in osteogenesis remain largely unknown. In the present study, we showed that RSPO3-shRNA increased the osteogenic potential of human adipose-derived stem cells (hASCs) significantly. Mechanistically, we demonstrated that RSPO3 is a negative regulator of ERK/FGF signalling. We confirmed that inhibition of the ERK1/2 signalling pathway blocked osteogenic differentiation in hASCs, and the increased osteogenic capacity observed after *RSPO3* knockdown in hASCs was reversed by inhibition of ERK signalling. Further, silencing of *LGR4* inhibited the activity of ERK signalling and osteogenic differentiation of hASCs. Most importantly, we found that loss of LGR4 abrogated RSPO3-regulated osteogenesis and RSPO3-induced ERK1/2 signalling inhibition. Collectively, our data show that ERK signalling works downstream of LGR4 and RSPO3 regulates osteoblastic differentiation of hASCs possibly via the LGR4-ERK signalling.

Human adipose-derived stem cells (hASCs) represent a readily available, abundant supply of mesenchymal stem cells, which are capable of self-renewal and differentiation into cells such as osteoblasts, chondrocytes and adipocytes^1^,^2^,^3^. As a potential cell source for bone tissue engineering, hASCs have attracted much attention^3^,^4^. To improve the osteogenic differentiation of hASCs effectively in bone tissue engineering, it is crucial to gain a better understanding of the molecular mechanism underlying the differentiation of hASCs. Osteogenesis is defined by a series of events, which starts with a commitment to an osteogenic lineage by mesenchymal stem cells. Subsequently, these cells proliferate, accompanied by an upregulation of osteoblast-specific genes and mineralization^3^. Multiple signalling pathways, including transforming growth factor β/BMP, Wnt/β-catenin, Notch, fibroblast growth factor (FGF), and Hedgehog, participate in the differentiation of an osteoblast progenitor to a committed osteoblast^5^,^6^,^7^,^8^,^9^,^10^. In particular, FGFs are important molecules that control bone formation. FGFs act by activating FGF receptors (FGFRs) and downstream signalling pathways that control cell differentiation along the osteoblastic lineage. Recent studies revealed that ERK1/2 signalling was induced by FGF2 to promote the proliferation of osteoblast precursors cells^11^. Additionally, ERK1/2 signalling mediates osteogenic differentiation of mesenchymal stem cells, induced either by FGF18^12^ or by activation of FGFR2 mutation^13^. It is well established that FGF promote osteogenic differentiation of mesenchymal stem cells through the ERK1/2 signalling pathway^14^.

R-spondins are a group of four highly related secreted proteins (RSPO1–4) with critical roles in development, stem cell survival, organogenesis and oncogenesis^15^,^16^,^17^,^18^. One of the family members, R-spondin 3 (RSPO3), has an important function in placental development, endothelial and blood differentiation, and malformation of head cartilage^19^. Mammalian RSPO3 contains two furin-like cysteine-rich (FU) domains near the N-terminus, a thrombospondin type I (TSP1) domain in the central region and a positively charged C-terminal region^17^. Knockdown of *rspo3* causes ventral oedema and vascular defects in Xenopus^20^. Rspo3-null mice suffer from severe vascular defects and are embryonic lethal^21^. Recently, R-spondins were identified as ligands of the leucine-rich repeat-containing G-protein coupled receptors (LGRs), including LGR4, 5 and 6^14^,^15^,^21^. RSPO-LGR was demonstrated play critical roles in development and stem cell survival. However, the exact roles of this ligand-receptor system in osteogenesis remain largely unknown.

In the present study, we first identified that RSPO3 is a negative regulator of hASCs osteogenic differentiation. *RSPO3* silencing leads to activation of ERK signalling pathway, which is essential for osteoblast differentiation of hASCs. LGR4 positively regulates osteoblast differentiation of hASCs via ERK signalling pathway. Moreover, loss of LGR4 attenuates the enhanced osteogenesis induced by *RSPO3* silencing. Together, our findings suggested that RSPO3 functions as a negative regulator of osteogenesis possibly through a LGR4-ERK dependent mechanism.

## Results

### Downregulation of endogenous *RSPO3* increases the osteogenic differentiation of hASCs *in vitro*

**Figure 1:**
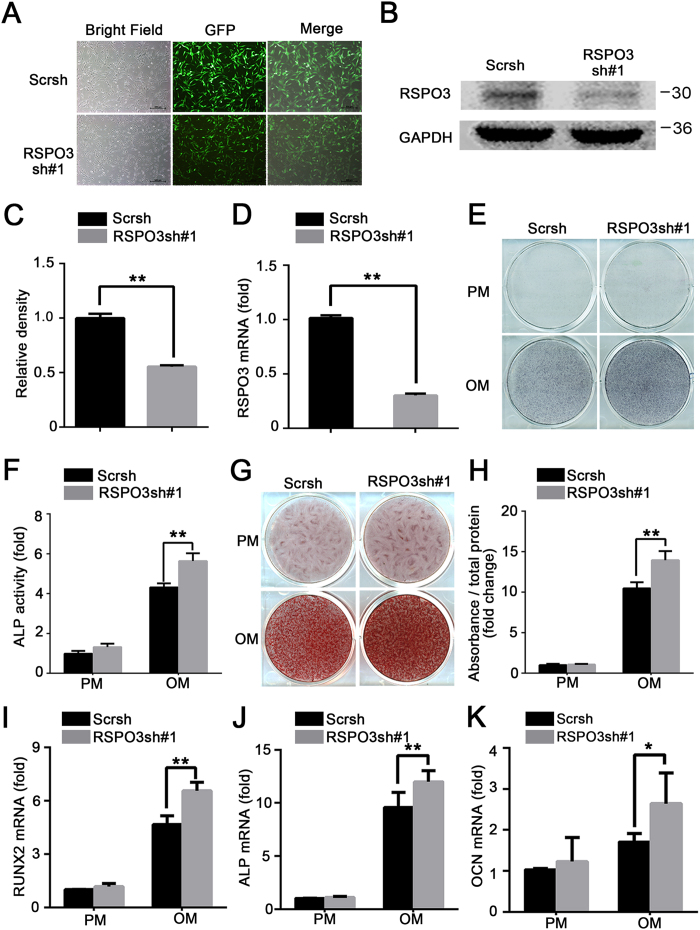
Knockdown of *RSPO3* increases the osteogenic differentiation of hASCs. (**A**) The knockdown efficiency of RSPO3 in hASCs was validated by fluorescence microscopy. Scale bar represents 100 μm. (**B–D**) Knockdown of *RSPO3* was validated by western blotting and RT-qPCR. (**E,F**) *RSPO3* knockdown increased the ALP activity in hASCs. Control or *RSPO3* knockdown cells were treated with proliferation or osteogenic media for 7 days for ALP staining (**E**), and cellular extracts were prepared to quantify ALP activity (**F**). (**G,H**) Knockdown of *RSPO3* increased mineralization of hASCs. Cells with or without *RSPO3* knockdown were treated with proliferation or osteogenic media for 14 days, and then calcium deposition was observed using Alizarin Red S staining (**G**) and quantification (**H**). The knockdown of *RSPO3* promoted the expression levels of *RUNX2* (**I**), *ALP* (**J**) and *OCN* (**K**) in hASCs, as assessed by RT-qPCR detection. *RUNX2* and *ALP* were detected at day 7 and *OCN* was detected at day 14 after osteoblast differentiation. All data are shown as the mean ± SD, n = 3. *P < 0.05 and **P < 0.01; PM: proliferation media; OM: osteogenic media.

To evaluate the potential role of RSPO3 in the process of osteogenic differentiation, we first investigated the expression of *RSPO3* in hASCs after osteogenic induction. As shown in Supplementary Fig. S1A,B, RT-qPCR showed that increased expression of *RSPO3* was accompanied by upregulation of the osteogenic marker *RUNX2*. We next generated a stable cell line with lentiviruses expressing an *RSPO3* shRNA. The knockdown efficiency was confirmed by immunofluorescence, western blotting, and RT-qPCR (Fig. 1A–D). In addition, we examined the expressions of *RSPO1, 2* and *4* by RT-qPCR after *RSPO3* silencing. There was no significant difference between the *RSPO3* knockdown cells and cells transfected with a scrambled shRNA (Supplementary Fig. S1C,D). After culturing the hASCs in osteogenic media (OM) for 7 days, alkaline phosphatase (ALP) activity was detected as being increased significantly by *RSPO3* knockdown (Fig. 1E,F). Moreover, the extracellular matrix mineralization, as determined by Alizarin Red S staining and quantification, was also augmented in *RSPO3* knockdown cells at 2 weeks after osteogenic induction (Fig. 1G,H). To confirm that *RSPO3* depletion promoted osteogenic differentiation, we investigated several osteogenic markers in osteogenically-stimulated hASCs. As shown in Fig. 1I–K, in contrast to the control cells, knockdown of *RSPO3* resulted in significantly increased mRNA expression levels of *RUNX2, ALP* and *OCN* (encoding osteocalcin). Furthermore, we investigated the proliferation levels of the *RSPO3*-silenced cells. The growth curve revealed that *RSPO3* silencing had no effects on the proliferation of hASCs, as determined by a CCK-8 assay (Supplementary Fig. S1E). In addition, the osteogenic differentiation of hASCs could also be blocked with another independent *RSPO3* shRNA fragment, but not with a random shRNA, excluding the possibility of off-target effects (Supplementary Fig. S2A–K). Taken together, these data indicated that downregulation of *RSPO3* promoted osteogenic differentiation *in vitro*.

### Overexpression of *RSPO3* inhibits the osteogenic differentiation of hASCs *in vitro*

**Figure 2:**
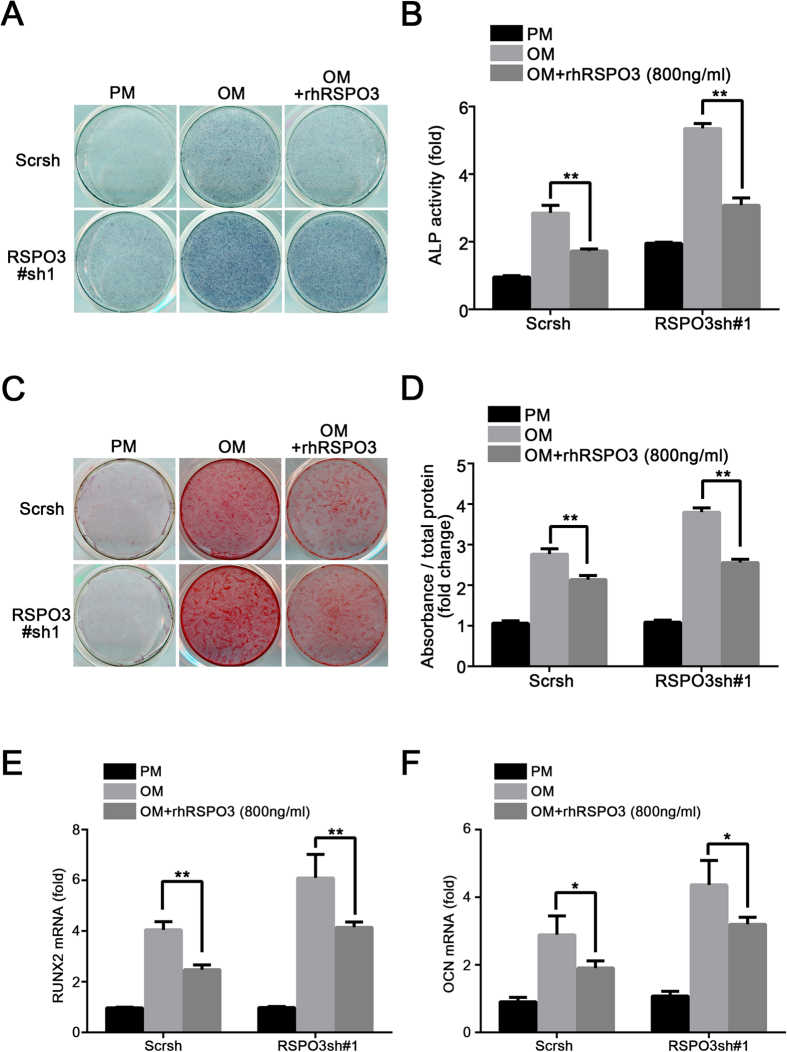
Recombinant human RSPO3 protein inhibits the osteogenic differentiation of hASCs *in vitro*. 800 ng/ml of rhRSPO3 was added to the osteogenic medium. (**A,B**) ALP staining and quantification of hASCs at day 7. (**C,D**) Alizarin Red staining and quantification of cells at day14. (**E,F**) rhRSPO3 inhibited the expression of *RUNX2* (**E**) at day 7 and *OCN* (**F**) at day 14 after osteogenic differentiation. All data are shown as the mean ± SD, n = 3. *P < 0.05 and **P < 0.01; PM: proliferation media; OM: osteogenic media.

To further confirm the important function of RSPO3 in osteogenesis, the recombinant human RSPO3 protein (rhRSPO3) was used for rescue experiments. At a concentration of 800 ng/ml, rhRSPO3 inhibited the upregulation of ALP activity and mineralization in RSPO3 knockdown cells as well as in Scrsh group cells (Fig. 2A–D). Consistently, treatment with rhRSPO3 resulted in decreased mRNA expression levels of *RUNX2* and *OCN* (Fig. 2E,F). Furthermore, we established *RSPO3* overexpression cells by lentivirus transfection in hASCs (Supplementary Fig. S3A-C). After osteogenic differentiation for 7 days, ALP activity was decreased in *RSPO3* overexpressing cells (Supplementary Fig. S3D-E), and extracellular matrix mineralization also decreased, as assessed by Alizarin red staining at day 14 (Supplementary Fig. S3F-G). In addition, RT-qPCR analysis revealed that *RSPO3* overexpression decreased *RUNX2* and *OCN* mRNA levels (Supplementary Fig. S3H-I). Taken together, these results indicated that RSPO3 inhibited osteogenic differentiation *in vitro*.

### Downregulation of *RSPO3* enhances hASC-mediated bone formation *in vivo*

**Figure 3:**
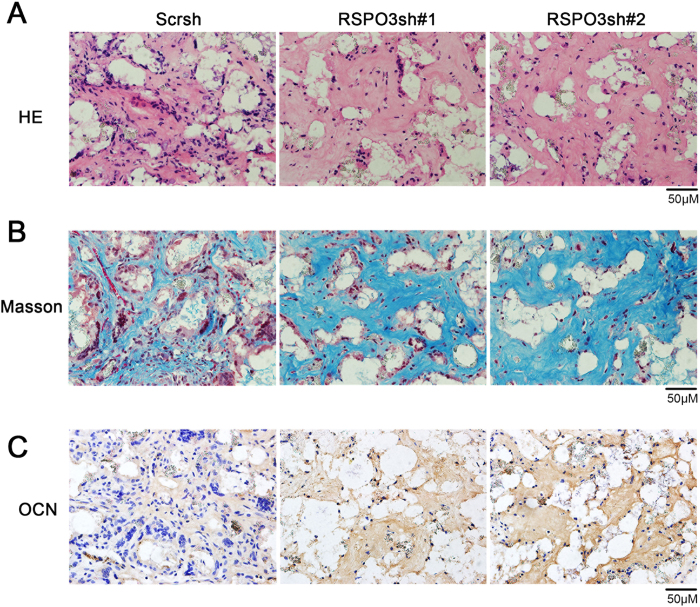
Knockdown of *RSPO3* promotes the osteogenic differentiation of hASCs *in vivo*. (**A**) Knockdown of *RSPO3* increased hASC-mediated bone formation *in vivo*, as determined by H&E staining of histological section from implanted hASC-scaffold hybrids. (**B**) Masson’s trichrome staining of histological sections from implanted hASC-scaffold hybrids. (**C**) Immunochemical staining for OCN. Scale bar represents 50 μm.

To verify our *in vitro* findings, we examine whether RSPO3 affected hASC-mediated bone formation *in vivo*. As shown in Fig. 3A, haematoxylin and eosin (H&E) staining showed that *RSPO3* knockdown cells formed more bone-like tissues compared with control cells. Quantitative measurements demonstrated that the area of bone formation was increased significantly in hASC/RSPO3sh cells (P < 0.05) (Supplementary Fig. S4A). Consistent with the observations from H&E staining, the osteogenic differentiation potential was markedly increased for hybrids containing hASCs/RSPO3sh compared with hASCs/Scrsh, as detected by Masson’ trichrome staining (Fig. 3B). Most importantly, we found that the osteogenic marker OCN was highly expressed in RSPO3sh cells, as determined by immunohistochemical (IHC) staining (Fig. 3C). Taken together, these results indicated that RSPO3sh hASCs could promote bone formation *in vivo*.

### Downregulation of endogenous *RSPO3* enhances ERK1/2 signalling pathway in hASCs

**Figure 4:**
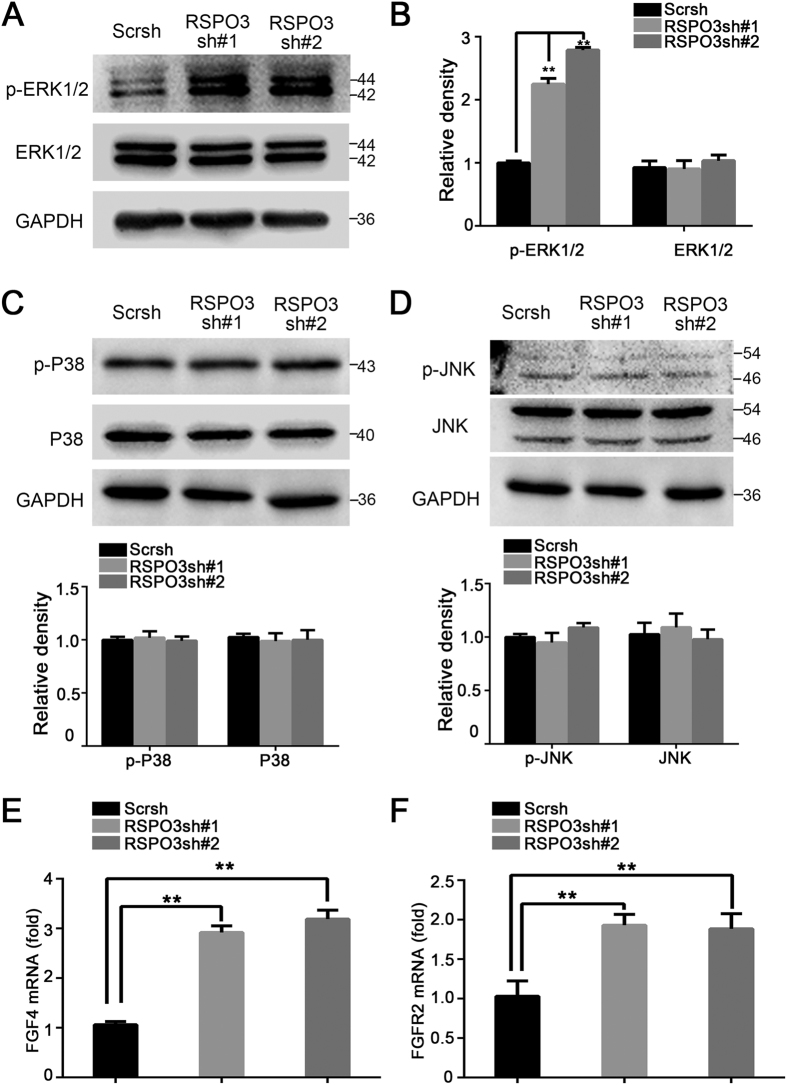
Downregulation of endogenous *RSPO3* enhances ERK1/2 activity. (**A**) Knockdown of *RSPO3* promoted phosphorylation of ERK1/2 in hASCs. Whole cell lysates were subjected to immunoblotting with the indicated antibodies. GAPDH was used as a loading control. (**B**) Quantitation of p-ERK and ERK expression levels. Immunoblots in (**A**) were scanned and normalized to GAPDH. (**C**) Cell lysates of control or *RSPO3* knockdown hASCs were subjected to immunoblotting with anti-p-P38 antibodies or anti-P38 antibodies. (**D**) Immunoblotting analysis showing the protein levels of p-JNK and JNK in control or *RSPO3* knockdown cells. (**E,F**) Knockdown of *RSPO3* promoted the expressions of *FGF4* (**E**) and *FGFR2* (**F**) in hASCs, as determined by RT-qPCR. *RSPO3* knockdown and control group cells were cultured in proliferation medium. All data are shown as the mean ± SD, n = 3. **P < 0.01.

To determine the molecular mechanism by which RSPO3 regulates osteogenic differentiation of hASCs, we screened several signalling pathways and key regulators of hASCs differentiation. Interestingly, we found that RSPO3 was responsible for the inhibition of ERK signalling in hASCs. We detected that the level of phosphor-ERK1/2 in RSPO3sh cells was increased significantly, as indicated by western blotting and immunoreactive band quantification (Fig. 4A,B). In contrast, the other MAPK cascades, p38 and JNK, were not affected by knockdown of *RSPO3* (Fig. 4C,D). It is well established that FGF promotes osteogenic differentiation of mesenchymal cell through the ERK1/2 signalling pathway. Therefore, we next examined the expression of FGF pathway genes in *RSPO3* knockdown cells. As shown in Fig. 4E,F, we observed increased *FGF4* and *FGFR2* gene expressions in RSPO3sh hASCs. This suggested that downregulation of endogenous *RSPO3* leads to activation of the ERK signalling pathway.

### Inhibition of ERK1/2 signalling pathway blocked osteogenic differentiation in hASCs

**Figure 5:**
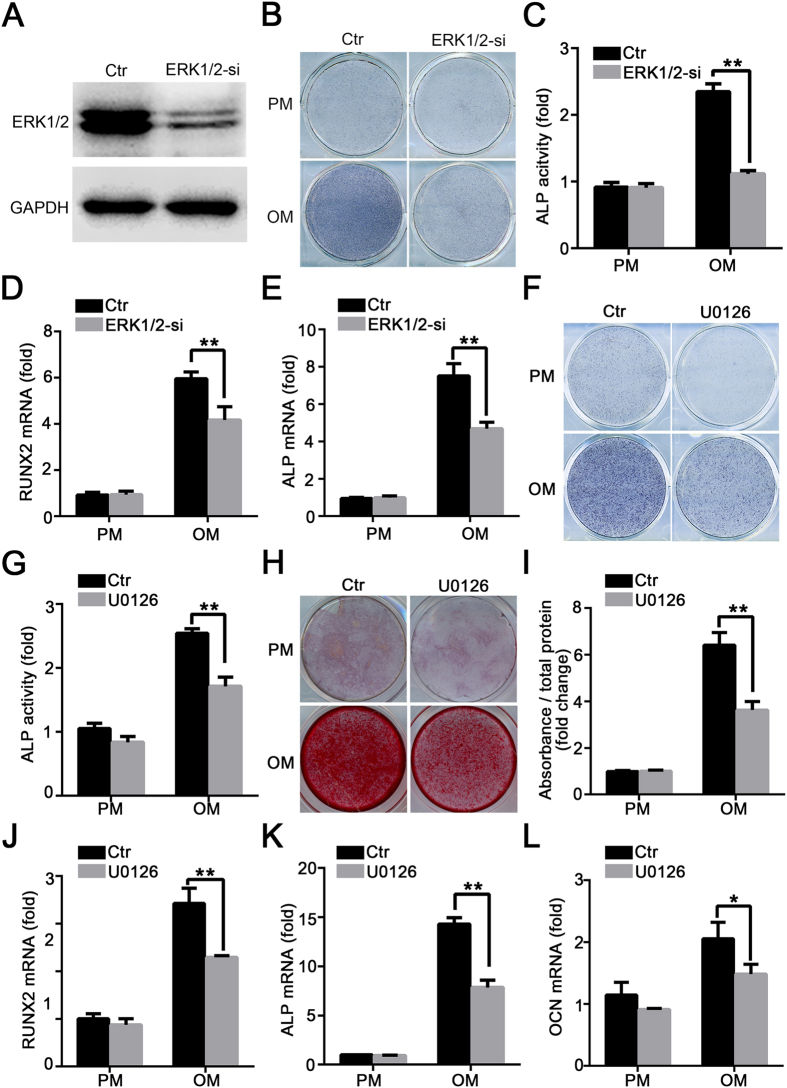
Inhibition of ERK signalling pathways inhibited osteogenic differentiation of hASCs. (**A**) Knockdown of ERK1/2 was validated by western blotting. Cells treated with proliferation medium were harvested after transfection with siRNA of *ERK1/2* for 48 h. (**B,C**) *ERK 1/2* knockdown decreased ALP activity in MSCs. Control or *ERK 1/2* knockdown cells were treated with proliferation or osteogenic media for 7 days for ALP staining (**B**), and cellular extracts were prepared to quantify ALP activity (**C**). (**D,E**) Knockdown of *ERK1/2* inhibited the expressions of *RUNX2* (**D**) and *ALP* (**E**) in hASCs, as determined by RT-qPCR. (**F,G**) U0126 treatment decreased ALP activity in hASCs. Cells were treated with proliferation or osteogenic media in the presence or absence of U0126 (10 μM) for 7 days for ALP staining (**F**), and cellular extracts were prepared to quantify ALP activity (**G**). (**H,I**) U0126 inhibited mineralization in hASCs. Cells in the presence or absence of U0126 (10 μM) were treated with proliferation or osteogenic media for 14 days, and then calcium deposition was observed using Alizarin Red S staining (**H**) and quantified (**I**). (**J–L**) U0126 treatment inhibited the expressions of *RUNX2* (**J**), *ALP* (**K**) and *OCN* (**L**) in hASCs as determined by RT-qPCR. *RUNX2* and *ALP* were detected at day 7 and *OCN* was detected at day 14 after osteoblast differentiation. All data are shown as the mean ± SD, n = 3. *P < 0.05 and **P < 0.01; PM: proliferation media; OM: osteogenic media.

To further determine the role of ERK1/2 in osteogenic differentiation of hASCs, we first analysed the activity of ERK1/2 after osteogenic differentiation. As shown in Supplementary Fig. S5A,B, the phosphorylation level of ERK1/2 was increased when cells were cultured in osteogenic media. Next, the small interfering RNA (siRNA) was used to knockdown the *ERK1/2* in hASCs. Silencing of *ERK1/2* expression decreased ERK mRNA and protein levels, whereas a control siRNA had no effect (Fig. 5A and Supplementary Fig. S5C,D). To further confirm the knockdown efficiency of *ERK1*/*2*, the expression level of ERK1/2 was evaluated in proliferation medium (PM) at 7 days and 14 days, separately (Supplementary Fig. S5E–J). As shown in Fig. 5B,C, in contrast to the control siRNA, the *ERK1/2* siRNA decreased ALP activity. Moreover, the extracellular matrix mineralization, as determined by Alizarin Red S staining and quantification, was also decreased in *ERK1*/*2*-silenced cells at 2 weeks after osteogenic induction (Supplementary Fig. S5K,L). In addition, *RUNX2* and *ALP* mRNA levels also decreased significantly in *ERK1/2* siRNA cells, as shown in Fig. 5D,E. These data indicated that ERK1/2 was a positive regulator of osteogenesis. To further confirm the role of ERK in the osteogenic differentiation of hASCs, we next investigated the effects of pharmacological inhibition of ERK1/2 by U0126 on osteoblast formation in OM. Upon treatment of hASCs with the ERK1/2 inhibitor U0126, the activity of ERK decreased significantly (Supplementary Fig. S5M-N). As shown in Fig. 5F,G, U0126 blocked the osteogenic capacity of hASCs, as indicated by ALP staining and quantification. Moreover, extracellular matrix mineralization, as determined by Alizarin Red S staining and quantification, was also impaired by U0126 treatment (Fig. 5H,I). Furthermore, U0126 decreased the expressions of *RUNX2, ALP*, and *OCN* in hASCs, as determined by RT-qPCR analysis (Fig. 5J–L). And most importantly, we detected that U0126 treatment had no effect on cell proliferation rate, as shown in Supplementary Fig. S5O. Collectively, the above data indicated the importance of ERK signalling in the osteogenic differentiation of hASCs.

### RSPO3 regulates osteogenic differentiation in an ERK-dependent manner

**Figure 6:**
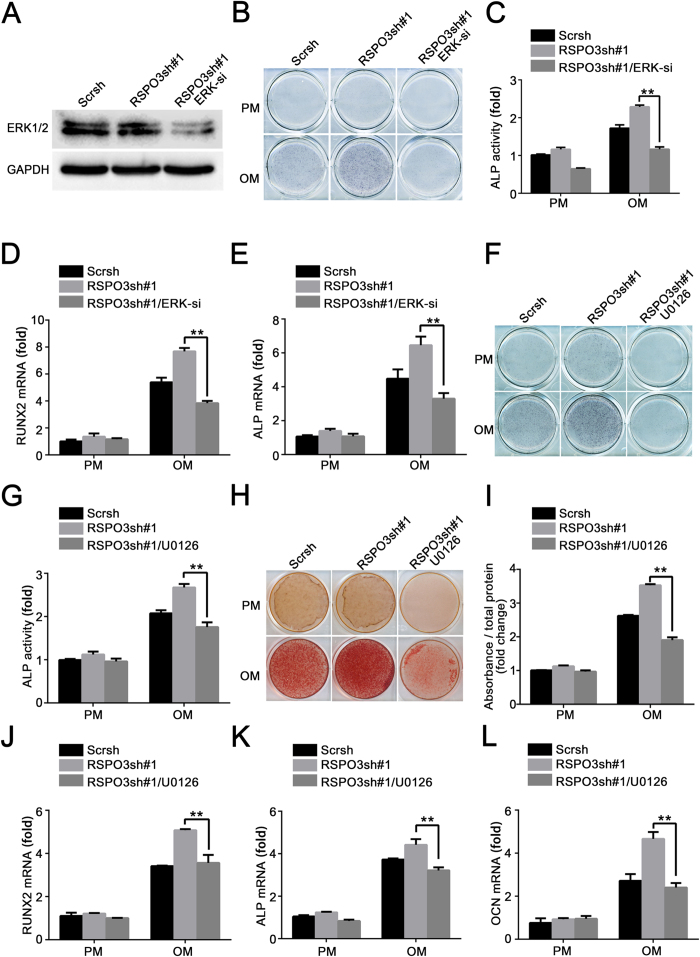
Inhibition of ERK1/2 signalling pathway abrogates the enhanced osteogenic differentiation of RSPO3sh hASCs. (**A**) Immunoblotting analysis showing the effective inhibition of ERK 1/2 in *RSPO3/ERK* double knockdown hASCs. Cells treated with proliferation medium were harvested after transfection with siRNA of *MAPK1/3* for 48 h. (**B,C**) ALP activity significantly decreased in *RSPO3/ERK* double knockdown cells compared with *RSPO3* knockdown cells, as determined by ALP staining (**B**) and quantification of ALP activity (**C**). *RUNX2* (**D**) and *ALP* (**E**) expression in the control, *RSPO3* knockdown and *RSPO3/ERK* double knockdown groups was determined by RT-qPCR. (**F,G**) Impaired osteogenic capacity of U0126 (10 μM) treatment in RSPO3sh hASCs is confirmed by ALP staining (**F**) and ALP activity assay (**G**) after 7 days of osteogenic induction. (**H,I**) U0126 treatment also inhibited mineralization of RSPO3sh hASCs. Control cells, *RSPO3* knockdown cells, and *RSPO3* knockdown cells in the presence of U0126 were treated with proliferation or osteogenic media for 14 days, and then calcium deposition was observed using Alizarin Red S staining (**H**) and quantified (**I**). (**J–L**) U0126 treatment abrogates upregulated *RUNX2* (**J**), *ALP* (**K**), and *OCN* (**L**) expression in RSPO3sh hASCs. *RUNX2* and *ALP* were detected at day 7 and *OCN* was detected at day 14 after osteoblast differentiation. All data are shown as the mean ± SD, n = 3. **P < 0.01; PM: proliferation medium; OM: osteogenic medium.

In light of the above observations, we hypothesized that inhibition of ERK1/2 signalling would abrogate the increased osteogenic capacity of *RSPO3* knockdown hASCs. To verify this hypothesis, two sets of osteogenic differentiation assays in RSPO3sh hASCs were performed either in the presence of U0126 or in the *ERK1/2*-silenced cells. As shown in Fig. 6A and Supplementary Fig. S6A, ERK1/2 was effectively knocked down in *RSPO3*-silenced cells, as determined by western blotting. When cells were treated with OM, the increased osteogenic differentiation ability induced by *RSPO3* knockdown was effectively reversed in the *RSPO3* and *ERK1/2* double knockdown cells, which was indicated by ALP staining and quantification (Fig. 6B,C). In addition, the increased expression of *RUNX2* and *ALP* caused by RSPO3 deficiency was also blocked by *ERK1/2* silencing (Fig. 6D,E). Next, we treated *RSPO3* knockdown cells in the absence or presence of U0126 in OM. As shown in Fig. 6F,G, U0126 decreased ALP activity, as determined by ALP staining and quantification. Additionally, Alizarin Red S staining and quantification was also inhibited in *RSPO3* knockdown cells treated with U0126 (Fig. 6H,I). Consistently, the increased expression of *RUNX2, ALP* and *OCN* induced by *RSPO3* knockdown was blocked in the presence of U0126 (Fig. 6J–L). Taken together, these results suggested RSPO3 regulates osteogenic differentiation in an ERK–dependent manner.

### Silencing of *LGR4* impaired the osteogenic differentiation potential of hASCs significantly

**Figure 7:**
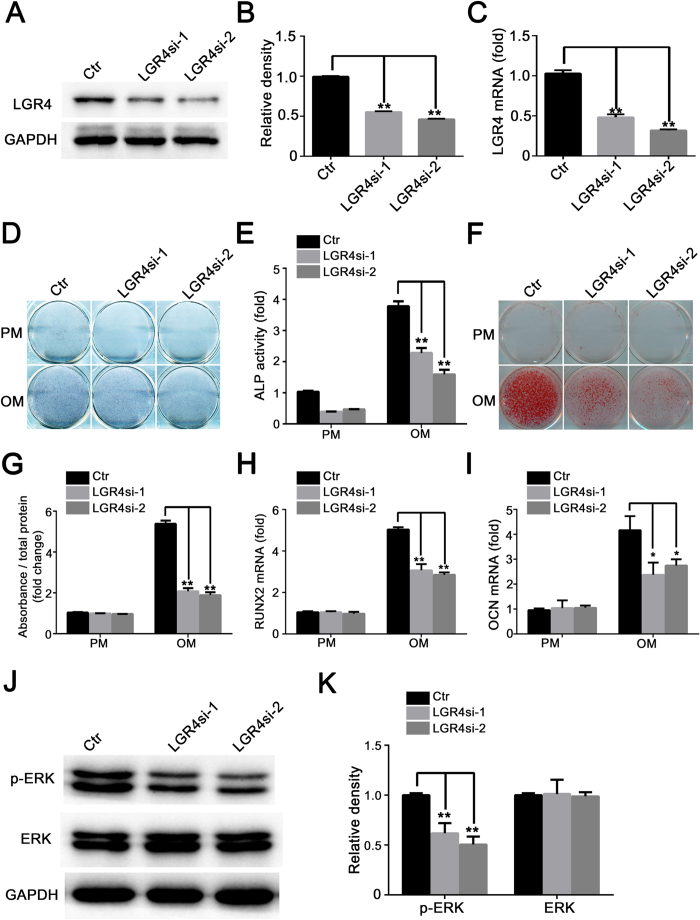
Silencing of *LGR4* impaired the osteogenic differentiation potential of hASCs. (**A–C**) Knockdown of LGR4 was validated by western blotting (**A,B**) and RT-qPCR (**C**). Cells treated with proliferation medium were harvested after transfection with siRNA of *LGR4* for 48 h. (**D,E**) *LGR4* silencing inhibited ALP activity of hASCs after osteoinduction for 7 days, as determined by ALP staining (**D**) and ALP quantification (**E**). (**F,G**) *LGR4* silencing inhibited the mineralization in hASCs after osteoinduction for 14 days. (**H,I**) RT-qPCR analysis of gene expression of *RUNX2* (day7) and *OCN* (day14). (**J**) *LGR4* silencing decreased the phosphorylation level of ERK1/2 in hASCs. (**K**) Quantification of p-ERK1/2 and ERK1/2 expression levels. Immunoblots in J were scanned and normalized to GAPDH. All data are shown as the mean ± SD, n = 3. *P < 0.05 and **P < 0.01; PM: proliferation medium; OM: osteogenic medium.

Previous studies demonstrated that R-spondins are *bona fide* ligands of the leucine-rich repeat-containing G-protein coupled receptors (LGRs), including LGR4, 5 and 6^15^,^16^,^22^. To investigate the potential role of LGRs on RSPO3-regulated osteogenesis, we first detected the expression levels of *LGR4*/*5*/*6* in the hASCs. As shown in Supplementary Fig. S7A,B, compared with *LGR4*, the expressions of *LGR5*/*6* were almost undetectable in hASCs. In addition, the expression level of *LGR4* decreased after osteogenic differentiation (Supplementary Fig. S7C). To determine the role of LGR4 in osteogenic differentiation of hASCs, we first conducted a small interfering RNA (siRNA)-mediated knockdown experiment. As shown in Fig. 7A–C, silencing of *LGR4* expression decreased the LGR4 mRNA and protein levels. To further confirm the knockdown efficiency of *LGR4*, we separately examined the LGR4 expression level after culturing for 7 days and 14 days in PM (Supplementary Fig. S7D–I). Next we detected the role of LGR4 in osteogenesis of hASCs. As shown in Fig. 7D–I, silencing of *LGR4* significantly decreased the ALP activity (Fig. 7D,E), mineralization deposit (Fig. 7F,G), and mRNA expression levels of *RUNX2* and *OCN* (Fig. 7H,I). In addition, the growth curves revealed that *LGR4* silencing had no effect on the proliferation of hASCs (Supplementary Fig. S7J). Most importantly, we found that LGR4 was a positive regulator of ERK signalling: the phosphor-ERK1/2 level in *LGR4*-silenced cells decreased significantly (Fig. 7J,K).

### Loss of *LGR4* abrogates RSPO3-regulated osteogenesis and RSPO3-induced ERK1/2 signalling inhibition

**Figure 8:**
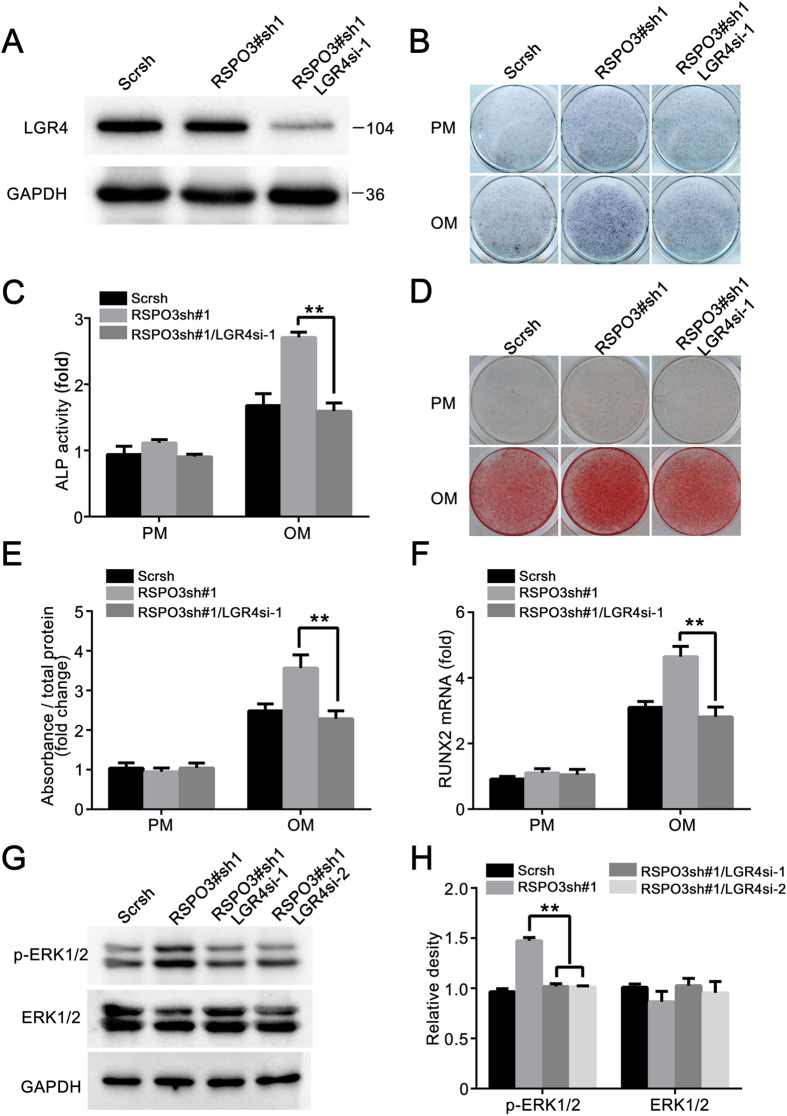
Loss of *LGR4* abrogates RSPO3-regulated osteogenesis and RSPO3-induced ERK1/2 signalling inhibition. (**A**) Double knockdown of RSPO3 and LGR4 was validated by western blotting. Cells treated with proliferation medium were harvested after transfection with siRNA of *LGR4* for 48 h. (**B,C**) Silencing of *LGR4* significantly decreased the *RSPO3* knockdown-promoted ALP activity, as determined by ALP staining (**B**) and ALP activity assay (**C**). (**D,E**) *RSPO3* knockdown-induced osteoblastic mineralization was attenuated after siRNA-mediated silencing of *LGR4* expression in hASCs, as determined by Alizarin Red staining (**D**) and quantification of mineralization (**E**). (**F**) The increased mRNA expression of *RUNX2* (day7) mediated by *RSPO3* knockdown was attenuated after functional disruption of LGR4 in hASCs. (**G**) Effects of *LGR4* knockdown on ERK activity in *RSPO3* knockdown hASCs. Whole cell extracts from control or *RSPO3* knockdown or *RSPO3/LGR4* double knockdown cells were subjected to immunoblotting with the indicated antibodies. (**H**) Quantification of p-ERK1/2 and ERK1/2 expression levels. Immunoblots in G were scanned and normalized to GAPDH. All data are shown as the mean ± SD, n = 3. **P < 0.01; PM: proliferation medium; OM: osteogenic medium.

We next examined the effect of *LGR4* siRNA on the osteogenic differentiation of *RSPO3* knockdown cells. As shown in Fig. 8A and Supplementary Fig. S8A, LGR4 was effectively silenced, as determined by western blotting analysis. When cells were treated with OM, the increase in osteogenic differentiation induced by *RSPO3* knockdown was effectively reversed in the *RSPO3* and *LGR4* double knockdown cells, which was indicated by ALP staining and quantification (Fig. 8B,C). Moreover, the extracellular matrix mineralization, as determined by Alizarin Red S staining and quantification, was also impaired in the *RSPO3/LGR4* knockdown cells compared with *RSPO3*-silenced cells (Fig. 8D,E). In addition, the increased expression of *RUNX2* caused by RSPO3 deficiency was also blocked by silencing of *LGR4* (Fig. 8F). These results suggested that LGR4 is involved in the RSPO3-regulated osteogenic differentiation. To further support this speculation, we next examined whether the knockdown of *LGR4* affected ERK signalling. As shown in Fig. 8G,H, western blotting demonstrated that knockdown of *LGR4* abrogated the increased level of phosphor-ERK1/2 in RSPO3sh cells. Taken together, these results indicated a novel role for RSPO3-LGR4 in osteogenesis by regulating the ERK signalling pathway.

## Discussion

In several genome-wide association studies to identify genes associated with osteoporosis, RSPO3 was demonstrated as a novel loci associated with bone mineral density(BMD) variations^23^,^24^,^25^. The present study demonstrated that RSPO3 plays an important role in osteogenic commitment of hASCs. When cultured in osteogenic medium, we found that RSPO3 deficiency could promote osteogenic differentiation of hASCs significantly, with increased ALP activity, matrix mineralization capacity, and mRNA expression of *RUNX2, ALP*, and *OCN*. Previous studies have shown that RSPO1 and RSPO2 could promote osteoblastic differentiation in synergy with Wnt proteins *in vitro*^26^,^27^,^28^,^29^. It appears that RSPO3 plays a different role in osteogenesis of hASCs. Investigation of the molecular mechanism suggested novel crosstalk between RSPO3 and the ERK1/2 signalling pathway. ERK1/2, a component of the MAPK signalling pathway, has been associated with cellular survival, proliferation and differentiation^30^,^31^. Despite numerous studies, the role of ERK1/2 in osteogenic differentiation is still a matter of some controversy. Administration of the ERK1/2 inhibitor PD98059 has been reported to promote early osteoblastic differentiation and mineralization in BMP2-treated C2C12 and MC3T3-E1 cells^32^,^33^. However, conflicting results obtained from other investigations indicated that activation of ERK signalling promotes osteogenic differentiation of stem cells in a cell-type specific manner^34^,^35^,^36^,^37^,^38^,^39^. In this study, we revealed the importance of the ERK pathway in the osteogenic differentiation of hASCs. To clarify the potential role of the ERK1/2, we established *ERK1/2* knockdown cells and showed that ERK1/2 deficiency significantly impaired the osteogenic differentiation of hASCs (Fig. 5A–E). Moreover, pharmacological inhibition of ERK1/2 with U0126 also impaired the osteogenesis of hASCs significantly (Fig. 5F–L). This work enriched our knowledge of the mechanisms underlying the regulation of hASCs osteogenic differentiation by the ERK signalling pathway. To further clarify whether ERK activation is necessary for increased osteogenic differentiation of *RSPO3*-silenced hASCs, we blocked the ERK1/2 signalling pathway using U0126 or an siRNA for *MAPK1/3*. The results showed that the increased osteogenic capability of *RSPO3*-knockdown hASCs was almost completely abrogated by U0126 treatment or *ERK1/2* silencing (Fig. 6F–L). Thus, ERK1/2 signalling is involved in the osteoblast differentiation of hASCs regulated by RSPO3. Despite the importance of ERK signalling in osteogenesis of hASCs, it is not the only pathway that is regulated by RSPO3. Numerous studies suggested that RSPO3 is associated with several important cell signalling and metabolic pathways. *Rspo3* deletion blocks Wnt/Ca^2+^/NFAT signalling by upregulation the expression of *Rnf213, Usp18*, and *Trim30α*, thus leading to the vessel regression phenotype of Rspo3-iECKO mice^40^. Deletion of RSPO3 leads to loss of SHH signalling and impaired organ growth in adrenal glands^41^. RSPO3 also controls metabolic zonation of liver hepatocytes via beta-catenin signalling^42^. Perhaps other factors are involved in RSPO3-mediated osteoblast differentiation of hASCs.

Notably, we observed that RSPO3 negatively regulates the ERK signalling. R-spondins were first discovered as secreted Wnt agonists^43^ and were identified as ligands of three related receptors LGR4–6^44^. LGRs bound with high affinity to the furin domains of R-spondins and then activated Wnt signaling^15^,^16^. Interestingly, our data showed that knockdown of *RSPO3* increased the phosphorylation of ERK1/2, while *LGR4* silencing exhibited opposite effect. *LGR4* silencing blocked the increased phosphorylation of ERK1/2 induced by *RSPO3* knockdown (Fig. 8G,H). LGR4 was identified as a key regulator of bone formation and resorption. Deletion of LGR4 in mice resulted in a dramatic delay in osteoblast differentiation and mineralization. LGR4 regulated bone formation and remodelling through the cAMP-PKA-Atf4 signalling pathway^45^. Silencing of *LGR4* abrogated the osteogenesis in MC3T3-E1 in presence of BMP2 or RSPO2^46^,^47^. These studies suggested that multiple signalling pathways were involved in LGR4-regulated osteogenesis, not merely ERK signalling. Recently, another two molecules have been validated as the ligands of LGR4. Norrin, a secreted protein, also a known ligand for Fzl4, stimulated Wnt signalling specifically through LGR4 but not LGR5 and 6. Norrin also interacted directly with Fzl4 to promote LRP5/6 internalization and to increase beta-catenin levels. Moreover, Norrin bound to BMP2/4, blocking the activation of type I and II BMP receptors, thus leading to decreasement in downstream Smad activities^48^. An intriguing and important idea is that the functions of Norrin and RPSO3 are similar: first, disruption of them led to vascular defect^20^,^21^,^49^,^50^,^51^; in addition, both RSPO3 and Norrin can bind to LGR4 and act as a Wnt agonist^15^,^16^,^48^. Until recently, studies about Norrin mainly focus on its biomedical effect in retinal vascularization. Considering the important function of Wnt and BMP signalling in osteogenesis, one cannot rule out the possibility that Norrin may be a strong competitor to RSPO3 in regulation of osteogenesis. Further studies are needed to verify the presumption. Additionally, it was also reported that LGR4 acted as a second receptor for RANKL. Luo, J. *et al*. showed that RANKL-LGR4-Gαq signaling promoted GSK3β activation, leading to NFATc1 nuclear export that downregulate NFATc1 expression and ultimately impairing osteoclastogenesis. Besides, LGR4 also competed with RANK for RANKL binding and attenuated RANK downstream signaling including NFκB pathway, calcium osscilation and AKT pathway. The study also demonstrated that RANKL competed with RSPO1 to interact with LGR4 in HEK293T cells, which prompted us that similar biological process may exist in hASCs^52^. In the present study, we confirmed that knockdown of *LGR4* inhibited the osteogenic differentiation of hASCs. Most importantly, we confirmed that LGR4 was involved in ERK signalling activation. Loss of *LGR4* abrogated RSPO3-regulated osteogenesis and RSPO3-induced ERK1/2 signalling inhibition. Although RSPO3 is reported as a ligand of LGR4, it is difficult for us to verify whether RSPO3 regulates ERK signalling directly through LGR4. There is a reasonable speculation that RSPO3 functions via LGR4 in regulating the activity of ERK signalling and osteogenesis, while some other related ligands and signalling pathways participate in this process, making it a complicated regulatory network.

In conclusion, we have shown that the RSPO3-LGR4 system plays an important role in osteogenic differentiation of hASCs. RSPO3 regulates osteogenesis possibly through the LGR4-ERK signalling pathway. This novel function of RSPO3-LGR4 might provide a new scientific rationale for the regulation of osteogenic differentiation of hASCs, and the regulatory control of ERK by RSPO3-LGR4 might extend beyond osteogenesis and have important implications in other cellular processes.

## Materials and Methods

All animal work in this study was approved by the Peking University Biomedical Ethics Committee Experimental Animal Ethics Branch, and was in accordance with the Guide for the Care and Use of the Laboratory Animals (National Academies Press, National Institutes of the Health Publication).

### Culture of hASCs

Primary hASCs were purchased from ScienCell Research Laboratories (Carlsbad, CA, USA). To induce osteogenic differentiation, hASCs were cultured in OM containing 100 mM/ml ascorbic acid, 2 mM β-glycerophosphate and 10 nM dexamethasone. All experiments were repeated three times using human ASCs from the three donors, respectively.

### Viral infection

Viral packaging and infection was prepared as described previously^53^. Briefly, HEK293T cells at 80% confluency were co-transfected with pLNB vectors with a mutant CBA promoter for gene expression or shRNAs, or plasmids psPAX2 (Addgene) and pVSV-G (Clontech, USA), using the PolyJet™ Transfection Reagent (SignaGen Laboratories, Rockville, MD, USA), according to the manufacturer’s instructions. The cell culture supernatant was collected at 36, 48 and 60 h after transfection, centrifuged and filtered through an Acrodisc filter with a 0.45-μm PVDF membrane (Pall Corporation, Port Washington, NY, USA) to remove cellular debris. The viral particles were then precipitated by centrifugation with PEG-it™ (System Biosciences, SBI, Mountain View, CA, USA). The concentrated lentiviral particles were titered, aliquoted and stored at −70 °C until ready for use. The lentivirus titre was 3 × 10^8^ TU/ml. Transfection of the hASCs was performed by exposing them to dilutions of the viral supernatant in the presence of polybrene (5 μg/ml) for 24 h. After 72 h of transfection, puromycin was used to select the stably transfected cells. The shRNA target sequences were: RSPO3sh#1, CCATTCATTCCCTGGCATTAA; RSPO3sh#2, GGCTACCTACAAGGTCAATTA.

### Alkaline phosphatase (ALP) staining

After 7 days of culture, cells were washed with PBS three times and then fixed in 4% paraformaldehyde at room temperature (RT) for 10 min. Subsequently, the cells were washed in PBS three times, incubated with a BCIP/NBT staining kit (CWBIO, Beijing, China) solution for 15 min at RT and rinsed with water.

### Quantification of ALP activity

After 7 days of culture, cells from six-well culture plates were washed three times with ice-cold PBS and then treated with 500 μl/well of 1% Triton X-100 (Sigma, St. Louis, MO, USA) for 5 min at RT for cell lysis. Cells were collected with a cell scraper, sonicated on ice and then centrifuged at 4 °C for 30 min at 12000 rpm. The supernatants were used to determine the protein concentration using a BCA protein assay reagent (Prod#23225, Pierce Thermo Scientific, Waltham, MA, USA) and to measure the ALP activity. ALP activity in cell lysates was measured using an ALP assay kit (A059-2, Nanjing Jiancheng Bioengineering Institute, China) and normalized by the total protein content.

### Alizarin Red staining and quantification

Analysis of mineralization was determined by Alizarin Red staining. Cells were induced for 2 weeks, fixed for 30 min in 70% ethanol at 4 °C and then rinsed with Milli-Q water. Calcium deposition was then visualized after incubation with 2% Alizarin Red S pH 4.2. Alizarin Red S was extracted by destaining with hexadecyl pyridinium chloride monohydrate. Mineral accumulation was quantified on a microplate reader at 562 nm and normalized by the total protein concentration detected in a duplicate plate.

### Quantitative real-time reverse transcription PCR (RT-qPCR)

Total RNA was extracted with the TRIZOL reagent (Invitrogen) and precipitated with ethanol. To exclude potential contamination of DNA, RNA was treated with DNase I for 30 min at 37 °C. cDNA was synthesized from 0.5–2 μg– of RNA with oligo (dT) 18 primers using a Quantscript RT Kit (Tiangen, Beijing, China).

RT-qPCR was performed on an Eppendorf Mastercycler ep realplex (Eppendorf, Hamburg, Germany) using the Fast-Start Universal SYBR Green Master Mix (Roche Applied Science, Mannheim, Germany). Reactions were carried out in a total volume of 20 μl, containing 2 μl of 1:10 diluted template cDNA, 10 μl of 2 × SYBR green PCR Master Mix (Roche Applied Science, Mannheim, Germany) and 100 nM of each primer. The following amplification program was used in all PCRs: 95 °C for 10 min, followed by 40 cycles of 15 s at 95 °C and 1 min at 60 °C. The specificity of each amplified reaction was verified by a dissociation curve (melting curve) analysis after 40 cycles, which was carried out by heating the amplicon from 60 to 95 °C. Moreover, the specificity of the PCR product was further confirmed by 2% agarose gel electrophoresis. Each sample was analysed in triplicate wells, and no-template controls (without cDNA in the PCR) were included. Data were collected and analysed quantitatively using realplex software.

The following forward and reverse primer sequences were used: *GAPDH* (internal control), (F) AAGGAGTAAGACCCCTGGACCA, (R) GCAACTGTGAGCAGGGGAGATT; *RSPO3*, (F) TGTCAGTATTGTGCACTGTGAGGT, (R) TCGGACCCGTGTTTCAGTCC; *RUNX2*, (F) ACTACCAGCCACCGAGACCA, (R) ACTGCTTGCAGCCT-TAAATGACTCT; *ALP*, (F) TGTGTGGGGTGAAGGCCAAT, (R) TCGTGGTGGTCACAATGCCC; OCN, (F) CACCATGAGAGCCCTCACACTC; (R) CCTGCTTGGACACAAAGGCTGC; *MAPK1*, (F) CCCATCGCCGAAGCACCATT, (R) TCCTCTGAGCCCTTGTCCTGA; *MAPK3*, (F) CATGGTCAGCTCGGCCTATGAC, (R) ATCTGGATCTCCCGGAGCGT; *FGF4*, (F) AGCTCTATGGCTCGCCCTTCT, (R) TCCCATTCTTGCTCAGGGCG; *FGFR2,* (F) GGTAACAGTTTCGGCTGAGTCCA, (R) TGCCGTTGAAGAGAGGCGTG.

### Bone formation *in vivo*

β-TCP (Bicon, Boston, MA, USA) was used for the *in vivo* study, according to our previous experiments^54^. Cells were cultured to 100% confluence *in vitro*, resuspended and incubated with β-TCP and then transplanted subcutaneously into the dorsa of nude mice. Two transplantation sites were prepared in each mouse, which were transplanted with three groups of cells: hASC/Scrsh, hASC/RSPO3 sh#1, and hASC/RSPO3 sh#2, as described previously. Transplants were harvested at 6 weeks and fixed in 4% paraformaldehyde. After decalcification in 10% EDTA (PH 7.4) for 10 days, the specimens were dehydrated and subsequently embedded in paraffin. Sections (5-mm thickness) were stained with haematoxylin and eosin (H&E) and Masson’s trichrome stain. Osteogenesis was evaluated by IHC analysis for OCN. For quantification of bone-like tissue, 10 images of each sample were taken randomly (Olympus, Tokyo, Japan) and SPOT 4.0 software (Diagnostic Instruments, Sterling Heights, MI, USA) was used to measure the area of new bone formation versus total area.

### Small interfering RNA

siRNAs targeting *MAPK1, MAPK3* and *LGR4* and negative control siRNA (NC-FAM) were obtained from Genepharma. siRNA sequences were: siMAPK1-1, CACCAACCAUCGAGCAAAUTT; siMAPK1-2, GUGCUCUGCUUAUGAUAAUTT; siMAPK1-3, CCACCUGUGAUCUCAAGAUTT; siMAPK3-1, GCUGAACUCCAAGGGCUAUTT; siMAPK3-2, ACACGCAGUUGCAGUACAUTT; siMAPK3-3, GACCGGAUGUUAACCUUUATT; siLGR4-1, GGUUGAAAGAACUCAAAGUTT; siLGR4-2, GAACUCAGCAUUUCACAAUTT; siLGR4-3, CACCUUUCAAGGCCUGAUATT. Cells at 70–80% confluence were transfected with 5 nM of siRNA using Lipofectamine RNAi-MAX (Invitrogen) and cultured for the indicated days. A mixture of siMAPK1-1, siMAPK1-2, siMAPK1-3, siMAPK3-1, siMAPK3-2 and siMAPK3-3 was used for ERK1/2 silencing. siLGR4-1 was used for LGR4si-1 as shown in Figs 7 and 8, Supplementary Fig. S7 and Supplementary Fig. S8. A mixture of siLGR4-2 and siLGR4-3 was used for LGR4si-2 as shown in Fig. 7 and Supplementary Fig. S7. Knockdown efficiency of siRNA was analysed by RT-qPCR and Western blot.

### Western blotting

Cells were harvested and then lysed in RIPA lysis buffer with a complete protease inhibitor mixture (Roche). Lysates were sonicated and centrifuged at 12000 rpm at 4 °C for 30 min to obtain the supernatant. The Pierce BCA protein assay kit (Thermo Scientific) was used to measure the protein concentrations. Aliquots (30 μg) of the protein extracts were subjected to 10% SDS PAGE and transferred to polyvinylidene difluoride membrane (Millipore). After blocking in 5% skim milk for 1 h, membranes were incubated with primary antibodies at 4 °C overnight, followed by incubation with peroxidase-linked secondary antibodies at room temperature for 1 h, and detection with an ECL kit (CWBIO) to visualize the immunoreactive protein bands. The antibodies used were anti-RSPO3 (Santa Cruz Biotechnology); anti ERK1/2, anti phospho-ERK1/2 (Thr202), anti SAPK/JNK, anti phospho-SAPK/JNK (Thr183/Tyr185), anti p38, anti phospho-p38 (Thr180/Tyr182) (Cell Signaling, Danvers, MA, USA); anti GAPDH, anti LGR4 (Abcam, Cambridge, UK).

### Inhibition of the activation of ERK by U0126

To block the activation of ERK signalling, U0126 (Sigma), a specific inhibitor of ERK activation, was added to the osteogenic differentiation medium for the entire 14-day differentiation period. The inhibiting effect was analysed by western blotting.

### Cell proliferation

The cell proliferation was examined with the CCK-8 (Cell Counting Kit-8) assay (Dojindo, Kumamoto, Japan) according to the manufacture’s protocol. At each time point, the supernatant of each group was removed, and cells were incubated in DMEM medium containing CCK-8 for 2 h at 37 °C. Optical density (OD) was measured at 450 nm using a microplate reader (ELX808, BioTek).

### Statistical analysis

All statistical analyses were performed using the GraphPad scientific software for Windows (San Diego, CA). Comparisons between two groups were analysed by independent two-tailed Student’s t-tests, and comparisons between more than two groups were analysed by one-way ANOVA followed by a Tukey’s *post hoc* test. Data were expressed as the mean ± standard deviation (SD) of 3–10 experiments per group. P values < 0.05 were considered statistically significant.

## Additional Information

**How to cite this article**: Zhang, M. *et al*. RSPO3-LGR4 regulates osteogenic differentiation of human adipose-derived stem cells via ERK/FGF signaling. *Sci. Rep.* **7**, 42841; doi: 10.1038/srep42841 (2017).

**Publisher's note:** Springer Nature remains neutral with regard to jurisdictional claims in published maps and institutional affiliations.

## Supplementary Material

Supplementary Information
