# Supplementary material for: RSPO3-LGR4 Regulates Osteogenic Differentiation Of Human Adipose-Derived Stem Cells Via ERK/FGF Signalling
Source: Sci Rep. 2017 Feb 21;7:42841. doi: 10.1038/srep42841 (PMC5318871; doi:10.1038/srep42841)

Supplemental Information

RSPO3-LGR4 regulates osteogenic differentiation of human adipose-derived stem cells via ERK/FGF signaling

Min Zhang[a,b1], Ping Zhang[a,b1], Yunsong Liu[a,b], Longwei Lv[a,b], Xiao Zhang[a,b], Hao Liu[b,c], Yongsheng Zhou[a,b,*]

a Department of Prosthodontics, Peking University School and Hospital of Stomatology, Beijing 100081, China

b National Engineering Lab for Digital and Material Technology of Stomatology, Beijing Key Laboratory of Digital Stomatology, Peking University School and Hospital of Stomatology, Beijing 100081, China

c Central Laboratory, Peking University School and Hospital of Stomatology, Beijing 100081, China

[1] These authors contributed equally to this work.

* Corresponding author: Yongsheng Zhou, D.D.S., Ph.D., Department of Prosthodontics, Peking University School and Hospital of Stomatology, 22 ZhongguancunSouth Avenue, Haidian District, Beijing100081, China.

Tel.: +86 10 82195370; fax: +86 10 62173402.

E-mail address: kqzhouysh@hsc.pku.edu.cn

Supplementary Fig. S1

(A-B) RT-qPCR analysis of *RPSO3* (A) and *RUNX2* (B) after osteogenic induction. (C-D) RT-qPCR analysis of *RSPO1*, *RSPO2* and *RSPO4* in *RSPO3* knockdown cells cultured in proliferation medium. (E) Growth curve of Scrsh and *RSPO3* knockdown hASCs. All data are shown as the mean ± SD, n = 3. **P < 0.01; PM: proliferation medium; OM: osteogenic medium.

Supplementary Fig. S2

(A-D) The knockdown efficiency of *RSPO3* in hASCs. (E–F) Control or RSPO3sh#2 cells were treated with proliferation or osteogenic medium for 7 days for ALP staining (E), and cellular extracts were prepared to quantify ALP activity (F). (G–H) Cells with Scrsh or RSPO3sh#2 were treated with proliferation or differentiation medium for 14 days, and then calcium deposition was observed using Alizarin Red S staining (G) and quantification (H). (I–K) Knockdown of *RSPO3* with RSPO3sh#2 promoted the expressions of *ALP* (I), *RUNX2* (J) at day 7 and *OCN* (K) at day14 in hASCs, as determined by RT-qPCR. All data are shown as the mean ± SD, n = 3. ***P* < 0.01; PM: proliferation medium; OM: osteogenic medium.

Supplementary Fig. S3

Overexpression of RSPO3 inhibited osteogenic differentiation of hASCs. (A-C) Expression of *RSPO3* determined by RT-qPCR (A) and western blotting(B-C). (D-E) RSPO3 overexpression decreased ALP activity in hASCs. Control or RSPO3

overexpression cells were treated with proliferation or osteogenic media for 7 days for ALP staining (D), and cellular extracts were prepared to quantify ALP activity (E). (F–G) Overexpression of RSPO3 inhibited mineralization of hASCs. Cells with or without RSPO3 overexpression were treated with proliferation or osteogenic media for 14 days, and then calcium deposition was observed using Alizarin Red S staining (F) and quantification(G). The overexpression of RSPO3 inhibited the expression levels of *RUNX2* (H), *OCN* (I) in hASCs, as assessed by RT-qPCR detection. All data are shown as the mean ± SD, n = 3. $^{**}P < 0.01$; PM: proliferation media; OM: osteogenic media.

Supplementary Fig. S4

(A) Quantitative measurements of bone-like tissues demonstrated that, at 6 weeks after implantation, the area of bone formation was significantly increased in RSPO3 knockdown cells compared with control cells. All data are shown as the mean ± SD, n = 3. $^{**}P < 0.01$

Supplementary Fig. S5

(A-B) The phosphorylation level of ERK1/2 in hASCs after osteogenic induction. (C) Quantification of ERK1/2 expression levels of Fig. 5A. Immunoblots in Fig. 5A were scanned and normalized to GAPDH. (D) The knockdown efficiency of *ERK1/2* in hASCs was validated by RT-qPCR. (E–J) The knockdown efficiency of *ERK1/2* in hASCs was analysed by western blotting and RT-qPCR at day 7 (E-G) and day 14 (H-J) in PM. (K-L) *ERK1/2* silencing inhibited mineralization of hASCs after 2 weeks of

osteogenic induction. (M-N) Whole cell lysates were subjected to immunoblotting with the indicated antibodies at 12h after treatment with the indicated concentrations of U0126. GAPDH was used as a loading control. (O) Growth curve of cells of the control and the U0126-treated group. All data are shown as the mean ± SD, n = 3. $^{**}P < 0.01$; PM: proliferation medium; OM: osteogenic medium.

Supplementary Fig. S6

(A) Quantification of ERK1/2 expression levels of Fig. 6A. Immunoblots in Fig. 6A were scanned and normalized to GAPDH. All data are shown as the mean ± SD, n = 3. **P < 0.01.

Supplementary Fig. S7

(A) The mRNA expression levels of *LGR4*, *LGR5*, and *LGR6* in hASCs were detected by RT-PCR. (B) RT-qPCR analysis of expression levels of *LGR4*, *LGR5* and *LGR6* in hASCs. (C) RT-qPCR analysis of *LGR4* after osteogenic induction. (D-I) The knockdown efficiency of *LGR4* in hASCs were analysed by western blotting and RT-qPCR at day 7 (D-F) and day 14 (G-I) in PM. (G) Growth curve of the control and *LGR4* silenced hASCs. All data are shown as the mean ± SD, n = 3. $^{**}P < 0.01$; PM: proliferation medium; OM: osteogenic medium.

Supplementary Fig. S8

(A) Quantification of LGR4 expression levels of Fig. 8A. Immunoblots in Fig. 8A were scanned and normalized to GAPDH. All data are shown as the mean ± SD, n = 3. **P < 0.01.

Supplementary Fig. S1

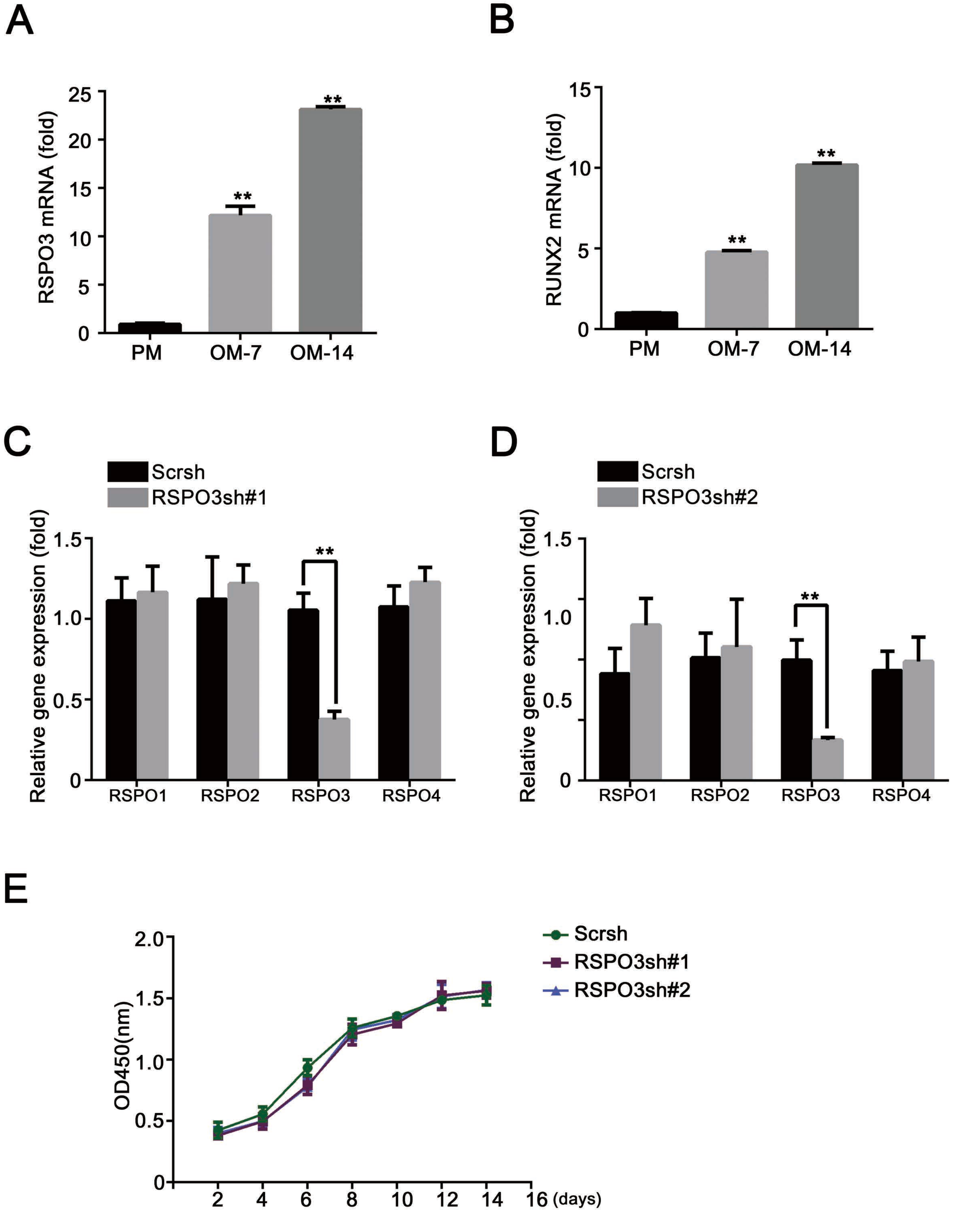

Supplementary Fig. S2

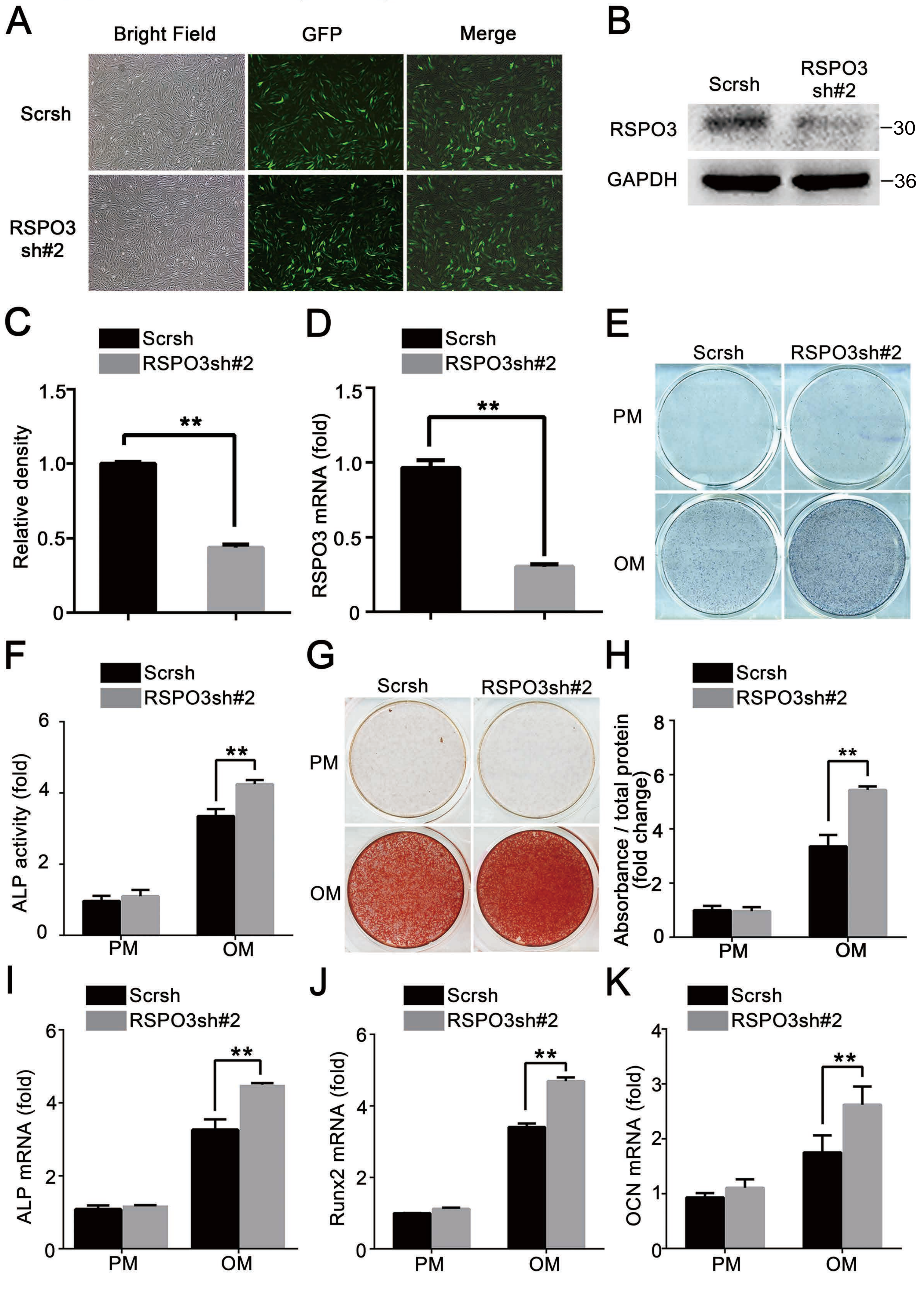

Supplementary Fig. S3

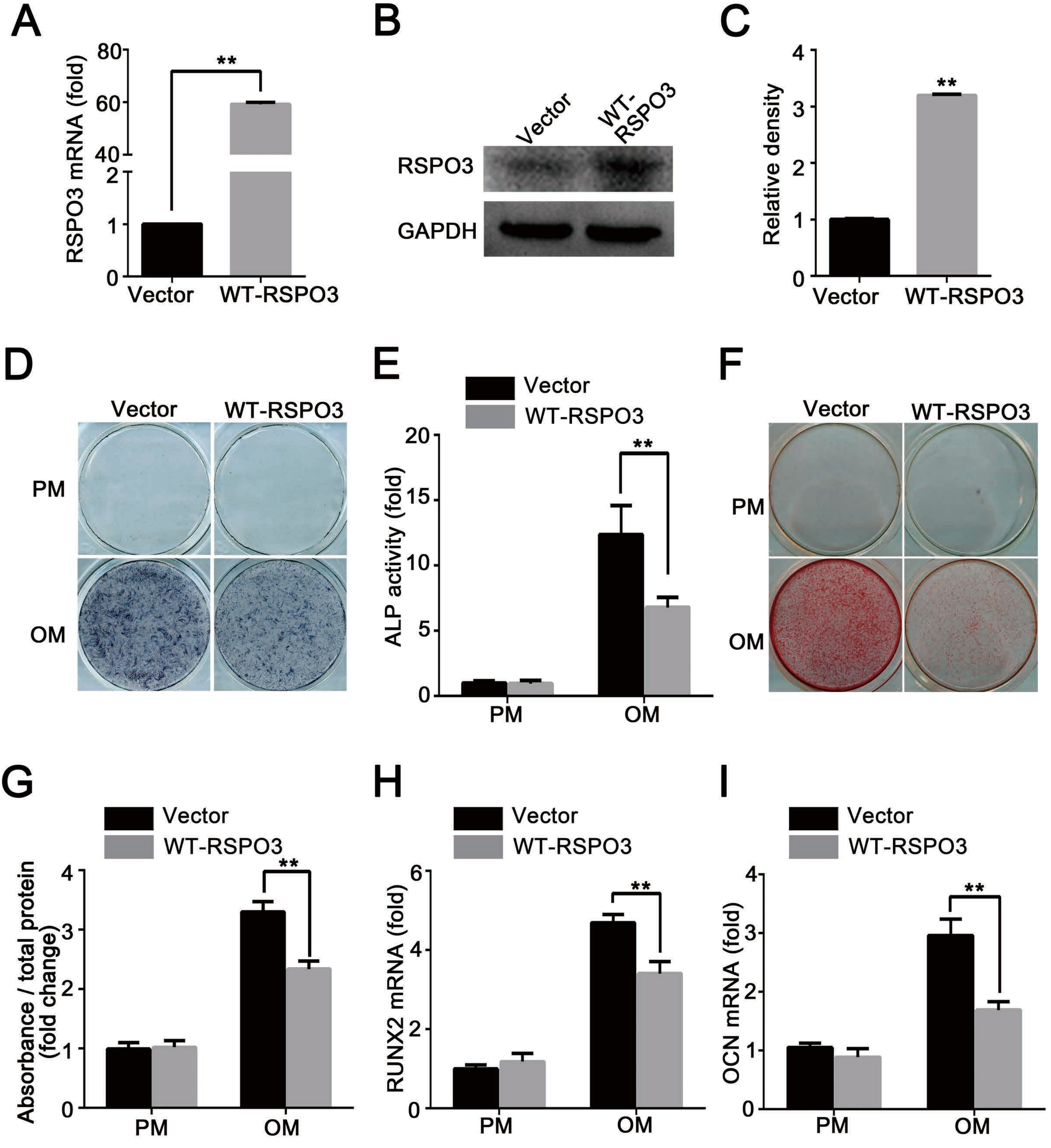

Supplementary Fig. S4

A

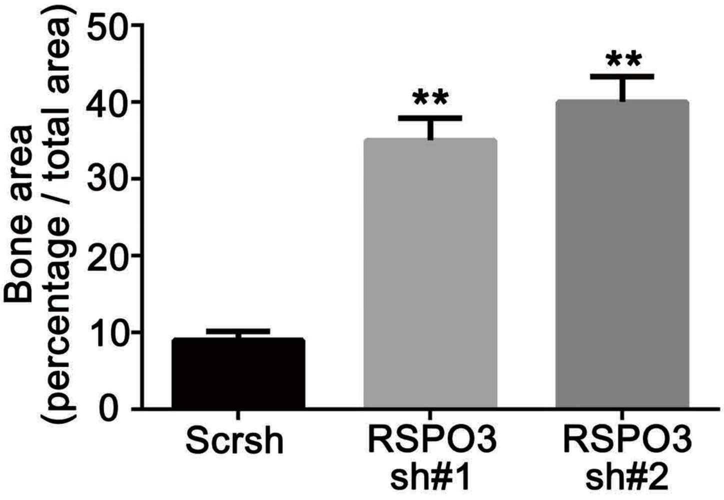

Supplementary Fig. S5

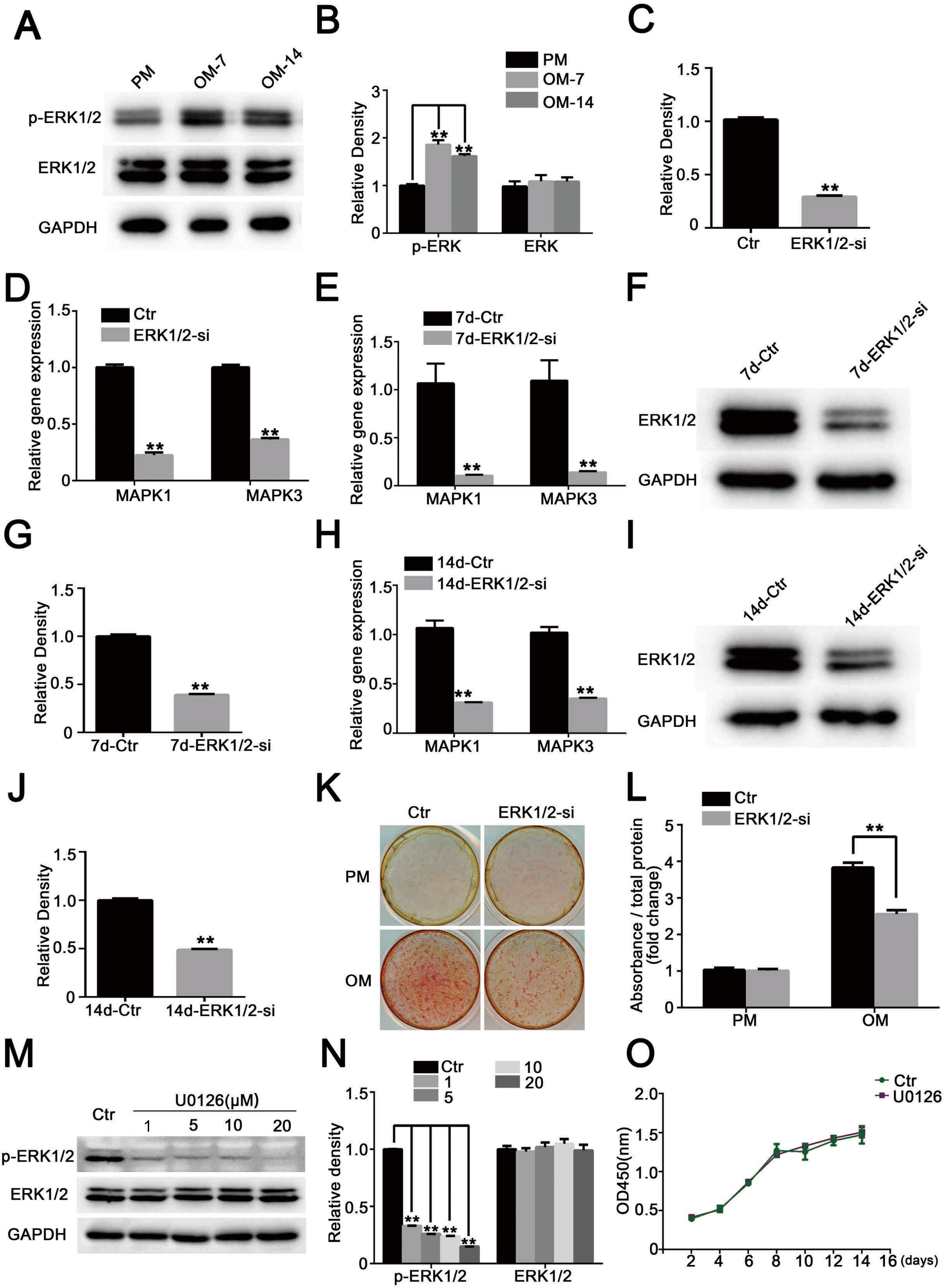

Supplementary Fig. S6

A

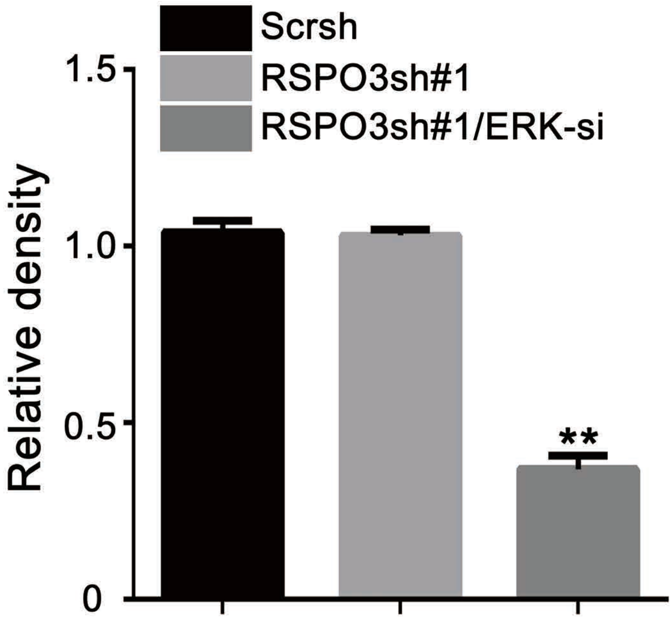

Supplementary Fig. S7

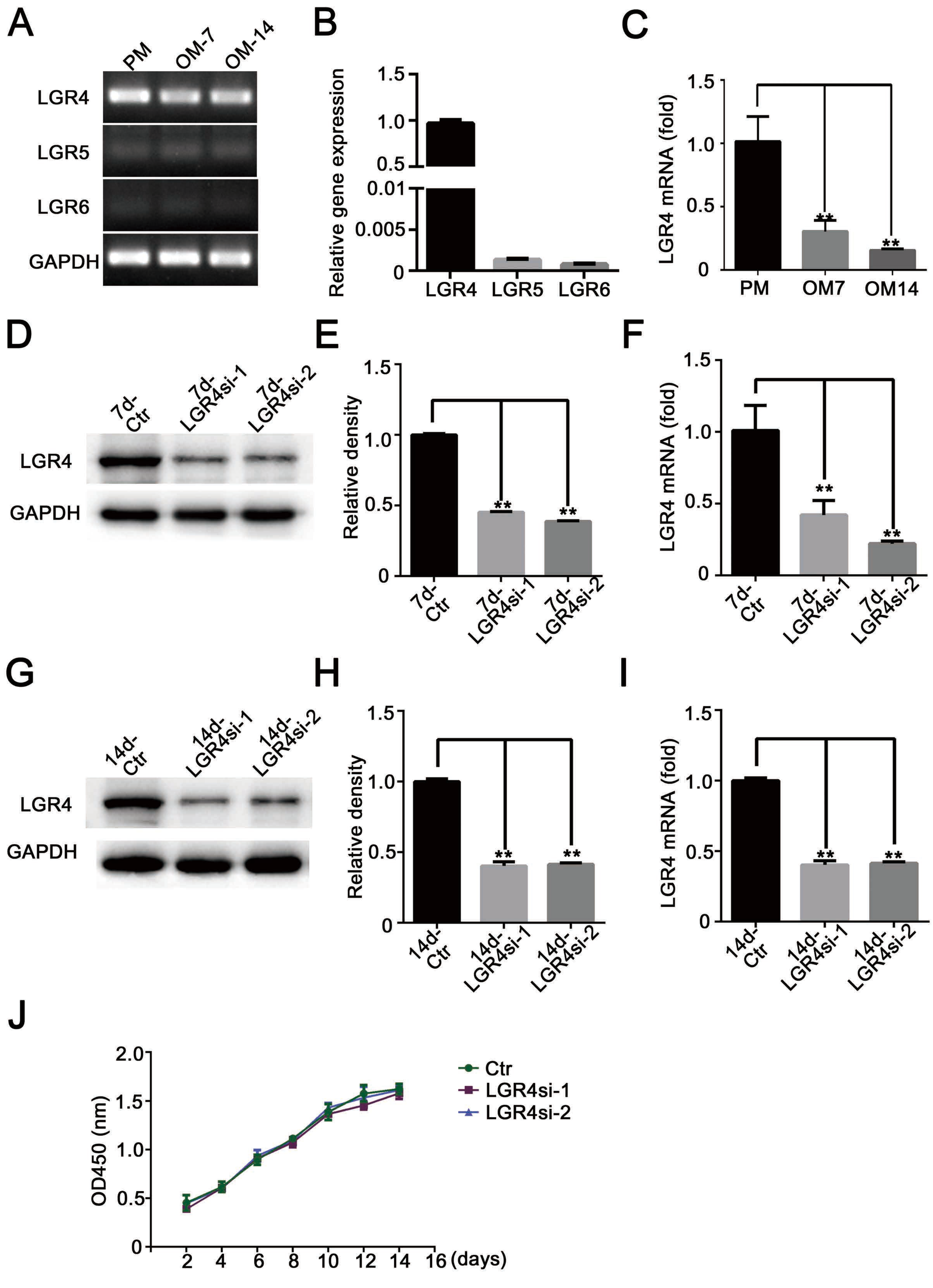

Supplementary Fig. S8

A

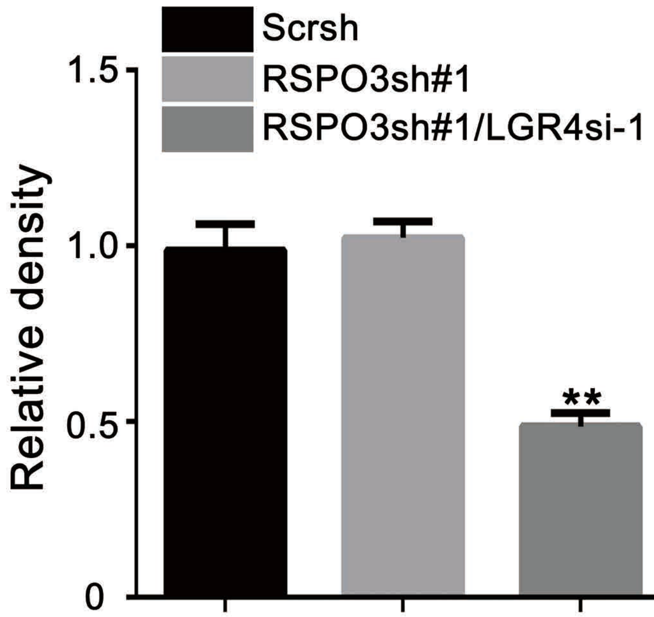

Supplement: Supplementary Information [file srep42841-s1.pdf]
